# Correction: Transcriptome-wide identification and expression profiling of the ERF gene family suggest roles as transcriptional activators and repressors of fruit ripening in durian

**DOI:** 10.1371/journal.pone.0260462

**Published:** 2021-11-18

**Authors:** The PLOS ONE Staff

There are errors in the Funding statement. The publisher apologizes for the errors. The correct Funding statement is as follows: This research was supported by the Ratchadapisek Somphot Fund for Postdoctoral Fellowship, Chulalongkorn University (to G.K.) and National Research Council of Thailand (NRCT5-RSA63001-15) and Chulalongkorn University (GRU 6407023008–1) (to S.S.).
