# Supplementary material for: Transcriptome-wide identification and expression profiling of the ERF gene family suggest roles as transcriptional activators and repressors of fruit ripening in durian
Source: PLoS One. 2021 Aug 10;16(8):e0252367. doi: 10.1371/journal.pone.0252367 (PMC8354473; doi:10.1371/journal.pone.0252367)
Supplement: S1 Table — (PDF) [file pone.0252367.s001.pdf]

S1 Table. List of primers for *DzERF*s and reference genes used in this study

Gene name	Forward primer (5'-3')	Reverse primer (5'-3')
DzERF6	TCGCCGTTATCACCACACC	TCCACGTCACCCATAACCCG
DzERF9	CGTCAGTTTAGTTTTGGGGTC	AACAGACTTAGAAGCAGGCCAT
**DzEF-1α*	GAAACCTTCTCTGCGTACC	CTCCACACTCTTGATGACAC
***DzACT*	AATGAGCAAAAAGGGTCAGCAC	GTCTTCAAAGTCAGCAGCCAG

* Durian elongation factor 1 alpha (first reference gene)

** Durian actin (second reference gene)
