# Supplementary material for: Transcriptome-wide identification and expression profiling of the ERF gene family suggest roles as transcriptional activators and repressors of fruit ripening in durian
Source: PLoS One. 2021 Aug 10;16(8):e0252367. doi: 10.1371/journal.pone.0252367 (PMC8354473; doi:10.1371/journal.pone.0252367)
Supplement: S1 Fig — Representative photos of three types of durian pulp samples (mature (unripe), midripe (∼3 days after harvest), and ripe (∼5 days after harvest)) during post-harvest ripening used in our study. (PDF) [file pone.0252367.s002.pdf]

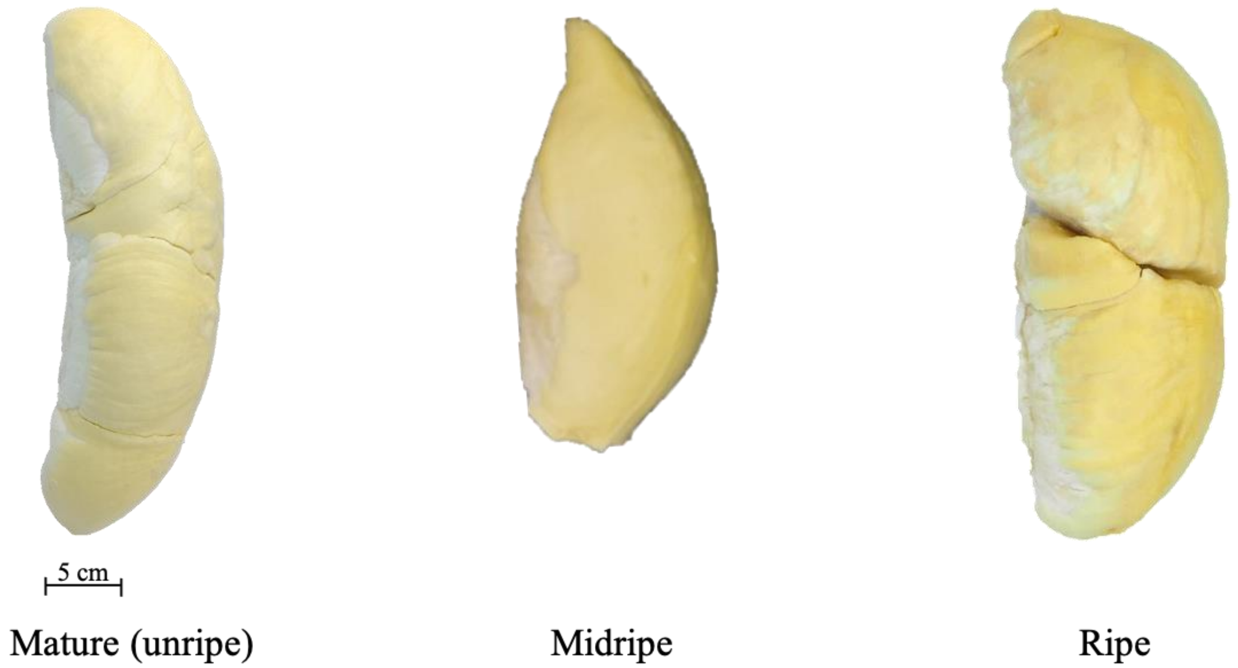

S1 Fig. Photos of durian pulp samples. Representative photos of three types of durian pulp samples (mature (unripe), midripe (~3 days after harvest), and ripe (~5 days after harvest)) during post-harvest ripening used in our study.
