# Supplementary material for: Transcriptome-wide identification and expression profiling of the ERF gene family suggest roles as transcriptional activators and repressors of fruit ripening in durian
Source: PLoS One. 2021 Aug 10;16(8):e0252367. doi: 10.1371/journal.pone.0252367 (PMC8354473; doi:10.1371/journal.pone.0252367)
Supplement: S2 Fig — Multiple sequence alignment analysis was carried out using ClustalW. A conserved DNA binding domain (DBD) of 61 amino acid residues designated the AP2/ERF domain was found at the N-terminal region of all DzERFs. Identical amino acids are highlighted by color. (PDF) [file pone.0252367.s003.pdf]

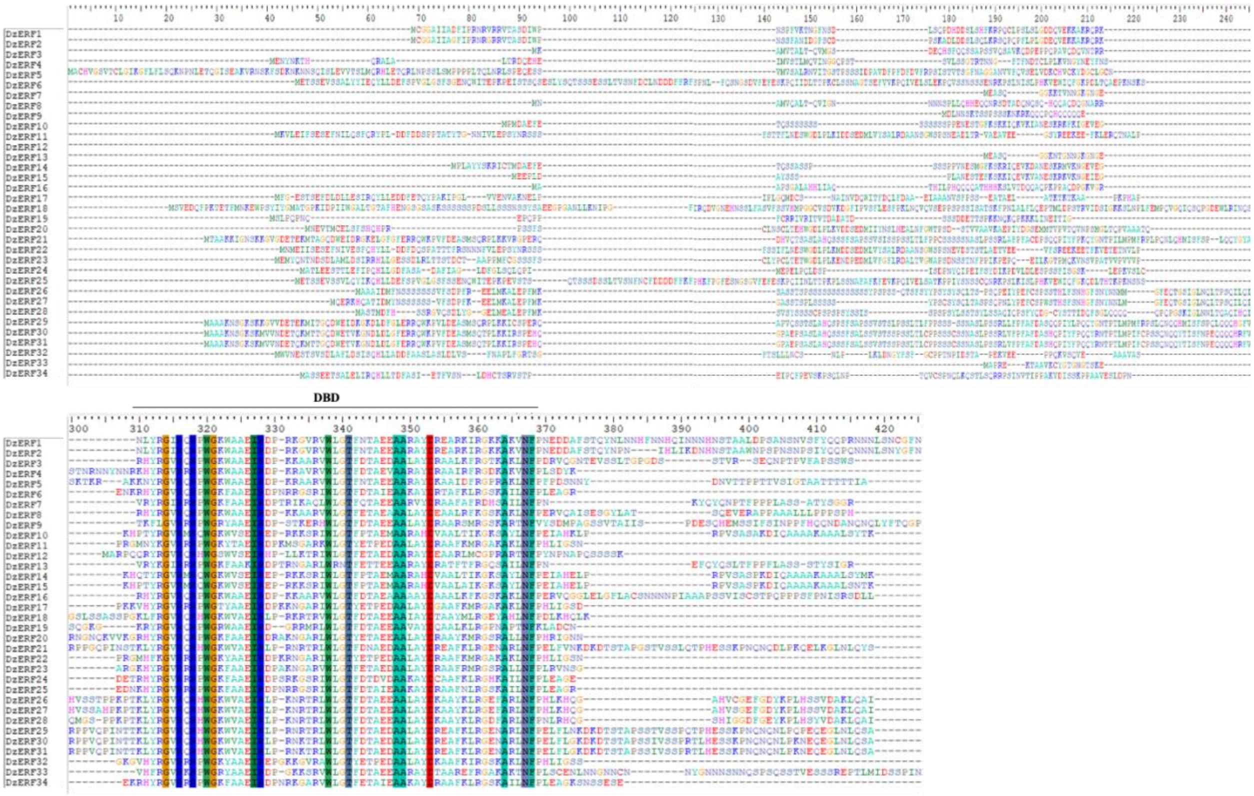

S2 Fig. Multiple sequence alignment of the amino acid sequences of the ripening-associated durian ERFs (DzERFs). Multiple sequence alignment analysis was carried out using ClustalW. A conserved DNA binding domain (DBD) of 61 amino acid residues designated the AP2/ERF domain was found at the N-terminal region of all DzERFs. Identical amino acids are highlighted by color.
