# Supplementary material for: Transcriptome-wide identification and expression profiling of the ERF gene family suggest roles as transcriptional activators and repressors of fruit ripening in durian
Source: PLoS One. 2021 Aug 10;16(8):e0252367. doi: 10.1371/journal.pone.0252367 (PMC8354473; doi:10.1371/journal.pone.0252367)
Supplement: S3 Fig — Protein sequences of ripening-associated DzERFs were used to identify conserved motifs. Ten conserved motifs were identified. Motifs 1 and 2 represent the conserved DNA binding domain (AP2/ERF domain) always observed at the N-terminus. (PDF) [file pone.0252367.s004.pdf]

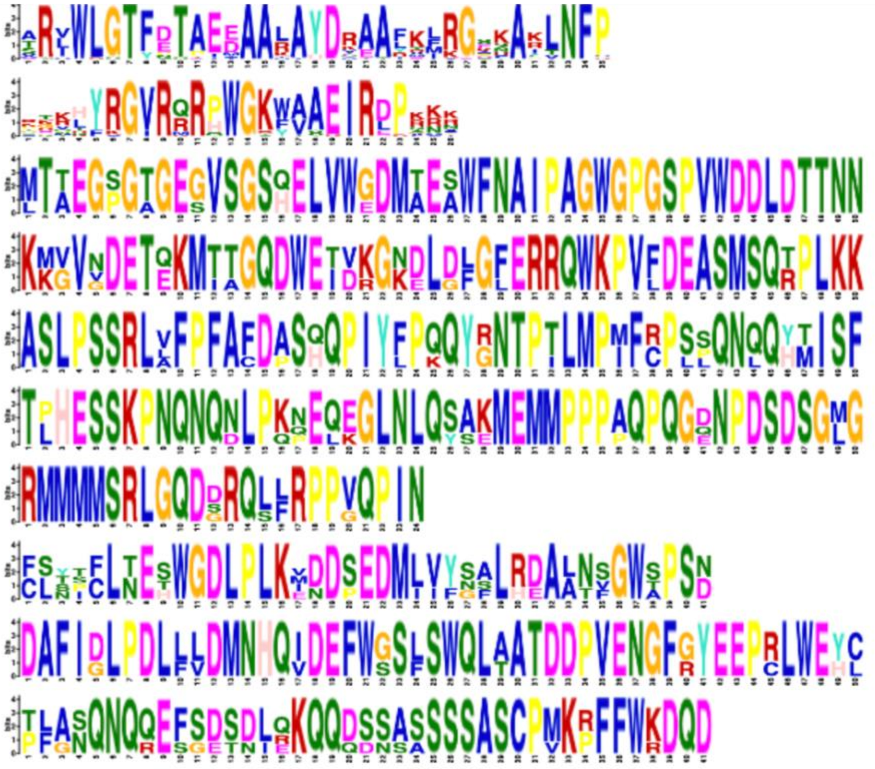

	Logo	E-value	Sites	Width
1.		7.6e-820	34	35
2.		1.4e-574	33	26
3.		6.6e-086	4	50
4.		2.2e-074	4	50
5.		1.6e-072	4	50
6.		1.7e-067	4	50
7.		1.3e-036	4	24
8.		4.8e-039	4	41
9.		5.4e-050	3	50
10.		1.5e-037	4	41

S3 Fig. Multilevel consensus sequences identified by MEME. Protein sequences of ripening-associated DzERFs were used to identify conserved motifs. Ten conserved motifs were identified. Motifs 1 and 2 represent the conserved DNA binding domain (AP2/ERF domain) always observed at the N-terminus.
