# Supplementary material for: Transcriptome-wide identification and expression profiling of the ERF gene family suggest roles as transcriptional activators and repressors of fruit ripening in durian
Source: PLoS One. 2021 Aug 10;16(8):e0252367. doi: 10.1371/journal.pone.0252367 (PMC8354473; doi:10.1371/journal.pone.0252367)
Supplement: S4 Fig — ERF binding sites: GCC box (AGCCGCC) and/or dehydration-responsive element/C-repeat (DRE/CRT) (CCGAC) are highlighted in yellow. The translational start site (ATG) is underlined. (PDF) [file pone.0252367.s005.pdf]

Durio zibethinus ACC synthase (*DzACS*)

CTTTGACCTGCAGAAAATTCTAATAATGCAGTAGATCTTTACGTTGACTTAAGTTTAGGATATAGCTAGC
CAATTGTTTGGACAATTCTCCTCTTACTTGGTTTCCTTATAAAAAGCTCATGTACAACTTGTTCTTTTAT
ATATAGTTAATCTTCTTTTTTCTAATACTCTTCCTATATCAGTTTTAGTCAATTGTTTTTTATTTAAAAA
CTATGCAGATTTGCCCGGGACCTAAGATGGCGCACTGGGTTAGAAATAGTTCCAGTTGACTGCAAAAGCT
CAAACAATTTTCGTATAACAAGAGCAGCCGCCGAAGAAGCCGCCGAAAAAGCTCAAAGATCAAACATCAA
TGTCAAAGGCGTGATCATAGCAAACCCCTCAAACCCTTTAGGCACAATCTTGGACAGAGAGACAATGAGA
AGCATAGTCAGTTTTATTAACGAACAGAACATCCACCTTGTCTCCGACGAAATCTATGCAGCTACAGTCT
TCAGCTCTCCTAGATTCATTAGCATTGCTGAGATTATACAAGATATGGATTGTAACCGTGATCTGATTCA
CATTGTTTACAGCTTGTCCAAGGACATGGGATTTCCTGGTTTTCGAGTTGGCATTGTTTACTCATTCAAT
GATGTAGTTGTGAATTGTGCCCGCAAGATGTCAAGTTTCGGATTAGTCTCCTCGCAAACTCAATACTTAC
TTGCTTCAATGCTTTCTGACGAGGATTTTGTTGGGAATTTCCTAAGGGAAAGCTCAAAGAGGTTAGCCGC
CCGGCACCATGTGTTCACCAAGGGACTCGAACAAGTGGGGATTTCTTGTCTAAAAAGCAATGCTGGTTTG
TTTTTCTGGATGGACATGCGACCCCTCCTTAAAGAACAAACTGTCAAGGGAGAAATGGAATTGTGGCGTG
TGATTATCGATGAAGTGAAACTCAATGTTTCTCCAGGTTCATCTTTCCAATGCTTGGAACCTGGCTGGTT
CAGGGTCTGCTTTGCAAACATGGATGGTGAGACCGTGGAAGTAGCACTTGACAGAATTCGAAAATTTGTG
CTTCAAGGAAAGGAAGAAGATCATGCGGTGCCAGAGACGTCAAAACGTTGGCAAAAGAAAAATCTTCGCC
TCAGCTTCTCTTCCTCTAGGTTATACGACGAGAGTATCATGTCTCCACCCATGATTTCCCCTCACTCCCC
GATACCTCACTCGCCCCTCGTTCCGGCGATGACTTGAGTAATAATTACAACGTAGCATTGCATGTCTCAT
CTTCGTAGTTCATCGTATTCATCTTTAATTAGTAATTTAAGTCATAAAAGTTCAATAGGAAATTCATTGT
CATAGATATCATTTTTAAGAGATTATTATGGCTCAGATTAATTTGGTACTTAAATCTTTTTGGTCTGACG
CCGACTTATTAAACCTCTTGATTTAGCCGCCAGGTGGAGAATAATAATCTTTTTCTCTAATTAATTTTTT
CCAAATTATAAAGTCTATGCTGTATGTAAGCATTATTTTTGTGGAGATATGCATTGTACTAGTAGTAATT
GTTCTATTCTAGGAGTCTTAGTTTGTACCAAGATGTTTGATTATAGTAATAATAATAATAAATGTGATTT
AGGATTCTAATTATTTTTATGAATTGAATTTGTGGTGTGCATCTAAAGCTTGAGGTCGCTGTGGAGAACA
CTAAGATTCTCAATTATCTTCATTCTTATGGGTTTTGATTTAACATTTTAATCACTAACTAAAGGTTAGC
TAAGTGGATGCTAAATAATCAATATGACATCATTGGATTGGCAATAACAGTATCCGTGCCTTCAATTTGC
TTTGGTGGAGCAAACATATAAATAAAACAGCTCAGACAAACAACCATGGCATGGAACATGATGTGATCAT
GGCATGTGCATTATGGATAAAACTATATGCAAATTTTCTTGGACATTTGTATATATACCCCATTTGAAAC
AGTGACTCAATGAAATTGAGAAGTTTTCAGGTCATGATCCATG

Durio zibethinus ACC oxidase (*DzACO*)

AAGGCAAAGGCACTTTAGGAAGTCTTTTATGATTTTTGGGTCATCAAAAAGTTGGTCTAATCCCACTCAA
AGAATCCAACCCAGGCCAAAGAGTGGATAGCCGCCTCAACAAGGCAAAGGGACTTTAGGAAGTCTTTTAT
GATTTTTGTTTCATTTGCTTTTACTATTATTTTCCCATTTCCTTTTTTTTTTTTTTTGTTAATTTTTAAC
TACATAATTTGCCTGACCCCCAAACCCTCCACTTGGAACTTCTATAAATACCACCACTCCTAGCTTCCAC
TCTGCAATCCGGAAATCTAAACTTTTGCGTAACACCCAAAGCAAGCGAGTGAAGACTTTGAGAGATTTTT
AGGAAAGAAAAAGAAAAGAGTGCTAAGAGAAATGGCAACTTTCCCAGTGATCTACATGGACAAGCTTAAT
GGTGAGGAGAGGGCAGCAACCATGGAGAAAATCAAGGATGCCTCTGAGAACTGGGGCTTCTTTGAGGTAT
CAAAAGGCTTAGCTATGCAGCTATGTACTATACTCTATTCTCCTCTACCTTTCTCCTTTATATTTTACTG
ATACGGTTATTTCTTCATTTCTTCCAGCTGCTGAACCATGGGATTCCCCATGATTTTCTGGACACTGTTG
AAAGATTGACAAAAGAGCATTACAAGAAATGCATGGAGCAGAGGTTTAAGGAACTGGTAGCAAGCAAGGC
CCTGGAGGGTCCCCAGGCAGAGGTGACTGATATGGATTGGGAGAGTACATTCTTCTTGCGCCATCTCCCT
GAATCAAACATGGCTGAAATTCCAGATCTCACTGATGAATACAGGTACAGAAGGAGTAAATTACAAGTTT
CAAAATGAAGTCAGATGTTCTAAATTCAATAAAAATAAATAAATTAGTCCCCTCCTAATGGTGCTTATTT
GGACAACAATAACTTTTCCCACTAAATTTCTTTCCCAGGGCTGTCTCCATAATTGGTTAATGCTGTCTCC
TCTTTCTTGACCAATGAATATAATATTGAATGATTTGACCATCATTGGCTTTTAGAATAATACCAACAAT
TACTTACTGCAATAAATTTTTCCCACTTTGTCTCTGTTTTGAAAACTTGCAAGTCCTGATACTTAGTCCA
AGTAAACAAAAATGCAGGAAGGTGATGAAAGAATTTGTACTGAAATTGGAGAAACTAGCGGAGGAGCTCC
TAGACCTGTTGTGTGAGAACCTTGGACTAGAGCAAGGATACCTGAAAAAGGCCTTCTATGGGGCAAGAGG
TCCAACCTTTGGCTCCAAAGTTAGCAACTACCCACCATGCCCAACCCCAGACAAAATCAAGGGACTCAGA
GCCCATACAGATGCAGGTGGCATCATCCTGCTCTTGCAAGACCCTAAAGTGAGCGGCCTCCAGCTTCTTA

AAGACGGGGAATGGATCGATGTTCCACCCAGCCGCCACTCCATTGTAATCAACCTTGGGGATCAGCTCGA
GGTATGCACTCTAAATCTTTACAATTTGCTTTTAGCTCAGTGCTTTTAGAAAGGAAAGTTAGACATCAAA
TTCTAAATCCTTAAACATACAGGTGATCACCAATGGCAAATACAAGAGTGTGGAGCACAGAGTGCTTGCC
CAAACTGACGGAGCTCGCATGTCTCTAGCTTCATTCTACAACCCTGGCAGGGATGCCCTTATCTACCCTG
CACCAGCTCTGGTGGAGAAAGAAGCAGAGGAGAAGAAACAATTGTACCCCAAATTTGTGTTTGAAGACTA
CATGAAGCTTTATGCTGGACTGAAATTCCAGGCCAAGGAACCAAGATTTGAAGCCGCCAAAGCCGCCGAA
ACAACTTTTCCCATTGCAACAGCTTAAAATTCTAGAGCTTCGTTTGACTTGATGGAGAAAAGGATGTGAT
CTATTTTCAACCTTTTAGTTGTGTGTGAAAGAAAAAAAAAAGAAGTCAAAGCTTACTGTAGGTGTGTGTT
GATATATTACTGTAAACAGCAACAACAATTCTATTCTACTATG

S4 Fig. Nucleotide sequences of the 2-kb promoter regions of ethylene biosynthetic genes from durian (*DzACS* and *DzACO*). ERF binding sites: GCC box (AGCCGCC) and/or dehydration-responsive element/C-repeat (DRE/CRT) (CCGAC) are highlighted in yellow. The translational start site (ATG) is underlined.
