# Supplementary material for: Transcriptome-wide identification and expression profiling of the ERF gene family suggest roles as transcriptional activators and repressors of fruit ripening in durian
Source: PLoS One. 2021 Aug 10;16(8):e0252367. doi: 10.1371/journal.pone.0252367 (PMC8354473; doi:10.1371/journal.pone.0252367)
Supplement: S5 Fig — Auxin response factor (ARF) binding sites (TGTCTC) are highlighted in yellow. The translational start site (ATG) is underlined. (PDF) [file pone.0252367.s006.pdf]

DzERF6

TATTTACCTTTGCACAGTGGAAAAACATGTTGCTTCCTCCTTCCTTCGTCCAGGCTTCGTTGAAAAGTTT
GAAAAGTCTTCAGATTCAACCTTCCTTGTAGCCGATAAATACCTTTCACTCTTCCCTTCCACTCTCAAGC
AAAAACTTTGAAAATCCTCTCTTCTTCAATTCAAACACACAATTCCAAAATCCTTTGCTCTTTTGTAATT
TTAAACTCTGAATTTTCTTTTTCCTTTCATTTTTGGTTATGGAAACAAGCAGTGAAGTGTCTCGCGCGCT
TTATTATATCGAACAATATTTACTTGATGAATTTTCTCCCGTGGGATTGGGGAGTTTTTCCGGTGAAAAT
CAATGGATAACTGAGCCAAAACCGGAAATTTCGACCTCTCAATCTGAGTCTCTGTACTCGCAAACTTCGA
GCTCTGAATCGTCTCTTACAGTATCCAATTTTGATTGCCTTAACGATGATGATTTCTTTAGATTCTCTCC
CAATTTGTTCCAGTCAAACGGAAGTGATGTTTTCGAATTTGAGTCGAAACCTCAAATCATCGATCTCACG
ACACCTAAGTGCCTCTCTTCGAACGCCGGTACTTCCGAATTTGTAGTCAAACCTCAAATCGTTGAACTTT
CACTGGAAAAACCTCAAGTTTCTTCGAATTCAAGCTCTGAGAACCGAAAACCGTCGCTTAATATCTCGTT
ACCGCATAAAGTGGAATGGATTCAGTTCGGCAAACCGGATTTAACTCAGGCGGAGCCGAAGAATTCAAAA
TCCGAAAACAAGAGGCATTATAGAGGTGTCTCACAACGTCCATGGGGGAAGTTCGCCGCGGAGATCCGAG
ATCCAAACCGAAGAGGTTCCCGGATCTGGTTAGGAACCTTTGACACTGCCATCGAAGCCGCAAAGGCTTA
CGACCGAACCGCGTTTAAGCTCCGTGGCTCGAAAGCGATTCTTAATTTCCCTCTTGAAGCCGGTAGGTTG
GATGCACACACCGTCGACGGAGAGAGGAAACGGAGCAGTGATGACCGCGAAGGAGAAGAGAGACAAGTGA
AGGCGGTGAAGAGAGACAATAATGACGTCACGAAATCGAGAGATAACGGTGATGTTCCTTTGACGCCGTC
AAATTGGAAATGCTTTTTGGATTGGGATAACGACGTGAAGGGGGTTTTTAACGTTCCACCACTGTCTCCG
TTATCACCACACCCGCCGTTAGGCTTTCCCCAAGTCATGGTTATATGATGATGAGAATGTGTCTCTTTGA
AGTGGCGGGTTATGGGTGACGTGGATTTCTTTGGGTTTAATATATAGTTGAATTGTTTTTAGAATTAGGC
AAGGATTTTTATTGGATTCAATTTCAATTTACTTGAATAAATTATTTTTTACATTTTTCATATGTGGCAT
GTTAGAAATTAAATTCTAATTATTAAAATTAAAATTTTAGATCACACTATTATTGTTTTTTTTTTTTTTC
ACTTTTATAGAATGTGTGAATCTTGATAAATTTAGCTTGAATCTATCCAAATTTATAAAATCAGTAGAAA
TTTTTTTTTATTTTCAAAATTATGATTATTTTTAATACATCGGATATAAAACAAATATGAATTATTTTTT
TATGAATAATTAAAATAAAAATTTTAGTTTTGTATGCAACAGTTATATTTGGGTATGTGATATATTTGAT
TATGAATTTTGTTAACAAGCATGCTACTGATATTTCTTTAATGTTGCTATATAAGTTAATATTTTTCATC
AAAAATCATCAATAAAATTATTAATTTTTACATTTAATAGAAAATTATTATTATAAATAATATGTAAGTT
AATATTTTTCATCGAAAATCATCAATAAATTTATTAATTTTTACATTTAATAGAAAATTATTATTATAAA
TAATATTAACTTAACTTTTACATTTAATAGAAAAATCATTATTTTAAATAATATTAACTAATATTAAAAA
TGTCTCATTTGTGAGCAAAGCCCTTTAACTATTTGCTTATATG

DzERF9

```
GTGCAGTTAACGCATATAATTTTGCTGCTCTACTTTTCTTACTCAAACATCATGGTATCATCACATAACA
AAAGTGCTTGTATTAATTGAGTGATTCATGATGAAGGGCAAAGGTGATCATGCGAGCTTATGAGGGTCCA
ATCAGAATCAAGGGCCCACGCTAGGATCATTAGCAAGCATTACTCACTCATAATTTGGATTAGAAAACCA
TGAATGCATTTGAAAATTTCTCCAAGGGCAACTCTGGTAAAATGGGGAGAATCTGATGGTGCAATCTCCT
TATTTAAACAACCAACATGCAAGCGGCCGAGTAACAAGGCCAACTATCATTCTTTTCTTAAAACCATCAA
TCTAATATTGCTCTATGCCATTTAGAGAAGACCAAGACTTCTCCCTTTTTACTCTCTTTCTGTCTCTCTA
AGGCAGGAAAAAAAAAAGAAGGAAAAAACTGCCCTTTAAAGCCCAGAGAGACGGACACTAAAAGAAAGGC
AGAAACACAAAAGTTTTTTCTTCGTCGCATTTAGCTGAAGCCTATCAACTCCAAAGCTTTCTTTATCTTC
ATGGATTTGAACAACTCAAAGACCAGCTCGCCTTCCTCTTCTAAAAACAAAAGAAAGCAGCAGCAACCTC
AGCACCAACAACAGCAACAAGAAACAAAATTCTTGGGAGTAAGGAGGAGGCCATGGGGTAGATACGCAGC
AGAGATAAGAGACCCTTCCACAAAAGAAAGGCATTGGCTTGGCACGTTTGACACTGCGGAGGAAGCAGCC
TTAGCCTATGACCGTGCCGCTCGCTCCATGCGTGGGTCCAAGGCTCGAACTAACTTTGTCTCTTCTGATA
TGCCAGCTGGCTCATCAGTCACAGCCATCATCTCCCCTGACGAATCACAGCATGAGATGTCTCCCATTTT
TTCCATTAATCCCCCTTTCCACCAACAAAACGACGCTAACCAGAACCAGCTTTACTTCACTCAAGGCCCT
TTCAATGCATGCCAGTTTTCTTCCGGGTTACCAGCTGGAGACGGGTGGAAGCAAGAGAGTGATACAGTTG
GGTCTTACAGGCCCATTACTGGTTTCATGGATGCAGCAAATGATGGTTCTAAACAGTTCAACGATGACTC
TGAGCTGCCACCTTTGCCTCCTGATGTCTCCTCTCTATGTTACGGCTCTGGAGTTGATGGTTCAGACATG
GGTTATGGAGCTTGGAATGACACAGGCCTTTTTGGGATCCCGGATCAAACTGCAAATGGGTTTGGCTCAG
GAGTCAGTGAACCGTATTTCGGCCTTAACTCTTACGAGTTCGTGCAGCACAGCCCACTCTTGAGGAGGAT
GCCATCTGTTCCTGATACGGTAGCTGACGGATTTGATTTGGGCTCGTCATCGGGTTACTTCTTTCAAGTA
CTGCCCCAAAATAAACAAAGCGCTACTAAAACTACATATTATCAGGACAATTGATTCGTCAGTTTAGTTT
TGGGGTCTTGCTGTTAATGGCTTTCTTATTTTGATGTTTTCGGCGGGACATAAGTAACGGTTTCTTGGGT
TGTCTCTGGATTTGGGTCCATTTTGGGAAAATGGCCTGCTTCTAAGTCTGTTTTATGGTTCATGTCTCGC
CTTTTGGTCAAAATTGTAGGGGGCGCGATGATAGCCCTTCTTTGGAGTGTAAAACTGAGGTGTGCAATGA
TTTTGATTTCTATAATCACCACGAGCTGCTTGGGGGCTTTTCTTCCGTTTTTCTTTTCTTTTAAACTTTA
AATAAAATCTGCCAACTCATACTTGTAAAAAAAAATTACAGTGTCTCACTAAGAGTCAATAATGTTTGAG
TGACTAACAATAAAAGTAATTACAATGAATAAATTATAAGAGGTAGGCCCCATTTGGATTTAACTTTTCC
ATCAGCATTGTGCGGTTAGGTTTTTTTTTTTTGGTGCGATTACGTGTTTTGGATAGTAATTAGTGCAATG
AAACACTTCACCGAAGCAATAATTTTAGACCGGAGCAAAAATG
```

S5 Fig. Nucleotide sequences of the 2-kb promoter regions of *DzERF6* and *DzERF9*. Auxin response factor (ARF) binding sites (TGTCTC) are highlighted in yellow. The translational start site (ATG) is underlined.
